# The effects of the Green-Mediterranean diet on cardiometabolic health are linked to gut microbiome modifications: a randomized controlled trial

**DOI:** 10.1186/s13073-022-01015-z

**Published:** 2022-03-10

**Authors:** Ehud Rinott, Anat Yaskolka Meir, Gal Tsaban, Hila Zelicha, Alon Kaplan, Dan Knights, Kieran Tuohy, Matthias Uwe Scholz, Omry Koren, Meir J. Stampfer, Dong D. Wang, Iris Shai, Ilan Youngster

**Affiliations:** 1 Faculty of Health Sciences, Ben-Gurion University of the Negev, 84105 Beer-Sheva, Israel; 2 BioTechnology Institute, University of Minnesota, Saint Paul, MN 55108 USA; 3 Department of Computer Science and Engineering, University of Minnesota, Minneapolis, MN 55455 USA; 4 Department of Food Quality and Nutrition, Research and Innovation Centre, Fondazione Edmund Mach, Via E. Mach 1, San Michele all’Adige, 38016 Trento, Italy; 5 University of Leeds, School of Food Science and Nutrition, Leeds, LS2 9JT UK; 6 Azrieli Faculty of Medicine, Bar Ilan University, Safed, Israel; 7 Brigham and Women’s Hospital, Harvard Medical School, Boston, MA USA; 8 Harvard T.H. Chan School of Public Health, Boston, USA; 9 The Health & Nutrition Innovative International Research Center, Faculty of Health Sciences, Ben-Gurion University of the Negev, Beer-Sheva, Israel; 10 Pediatric Division and Center for Microbiome Research, Shamir Medical Center, Be’er Ya’akov, Israel; 11 Sackler School of Medicine, Tel-Aviv University, Tel-Aviv, Israel; 12 Pediatric Infectious Diseases Unit and the Center for Microbiome Research, Shamir Medical Center, 70300 Zerifin, Israel

**Keywords:** Microbiota, Nutrition, Weight loss, Polyphenols, Meat, Mediterranean diet, Cardiometabolic health

## Abstract

**Background:**

Previous studies have linked the Mediterranean diet (MED) with improved cardiometabolic health, showing preliminary evidence for a mediating role of the gut microbiome. We recently suggested the Green-Mediterranean (Green-MED) diet as an improved version of the healthy MED diet, with increased consumption of plant-based foods and reduced meat intake. Here, we investigated the effects of MED interventions on the gut microbiota and cardiometabolic markers, and the interplay between the two, during the initial weight loss phase of the DIRECT-PLUS trial.

**Methods:**

In the DIRECT-PLUS study, 294 participants with abdominal obesity/dyslipidemia were prospectively randomized to one of three intervention groups: healthy dietary guidelines (standard science-based nutritional counseling), MED, and Green-MED. Both isocaloric MED and Green-MED groups were supplemented with 28g/day walnuts. The Green-MED group was further provided with daily polyphenol-rich green tea and Mankai aquatic plant (new plant introduced to a western population). Gut microbiota was profiled by 16S rRNA for all stool samples and shotgun sequencing for a select subset of samples.

**Results:**

Both MED diets induced substantial changes in the community structure of the gut microbiome, with the Green-MED diet leading to more prominent compositional changes, largely driven by the low abundant, “non-core,” microorganisms. The Green-MED diet was associated with specific microbial changes, including enrichments in the genus *Prevotella* and enzymatic functions involved in branched-chain amino acid degradation, and reductions in the genus *Bifidobacterium* and enzymatic functions responsible for branched-chain amino acid biosynthesis. The MED and Green-MED diets were also associated with stepwise beneficial changes in body weight and cardiometabolic biomarkers, concomitantly with the increased plant intake and reduced meat intake.

Furthermore, while the level of adherence to the Green-MED diet and its specific green dietary components was associated with the magnitude of changes in microbiome composition, changes in gut microbial features appeared to mediate the association between adherence to the Green-MED and body weight and cardiometabolic risk reduction.

**Conclusions:**

Our findings support a mediating role of the gut microbiome in the beneficial effects of the Green-MED diet enriched with Mankai and green tea on cardiometabolic risk factors.

**Trial registration:**

The study was registered on ClinicalTrial.gov (NCT03020186) on January 13, 2017.

**Supplementary Information:**

The online version contains supplementary material available at 10.1186/s13073-022-01015-z.

## Background

The Mediterranean (MED) diet, high in nuts, vegetables, and legumes and low in red meat intake, is recommended for the prevention of cardiometabolic diseases [1–3].

It has been reported that adherence to MED dietary patterns is associated with a distinct gut microbiome profile [4]. An 8-week study (*n* = 82) of an isocaloric MED diet intervention revealed changes in multiple microbial features in the gut, including an increased abundance of major dietary fiber metabolizers such as *Bacteroides cellulosilyticus* [5], concurrent with beneficial changes in cardiometabolic biomarkers [6]. However, to the best of our knowledge, no study has tested the degree to which the MED diet exerts its beneficial effects on cardiometabolic health through modulation of the gut microbiome.

While randomized controlled trials showed that dietary interventions targeting overall eating patterns lead to a substantial reduction in cardiometabolic disease risk [2, 7], optimizing dietary regimens known to promote health could have vast implications. Recently, we tested the effects of a modified MED diet, the Green-MED diet, which further increases the plant-sourced components while minimizing red and processed meat intake [8, 9]. The two key components of the Green-MED diet are daily intake of green tea adding large quantities of polyphenol compounds [10] and *Mankai*, a novel *Wolffia globosa* strain, providing additional polyphenols and plant-based protein.

We recently reported the beneficial effects of this modified MED diet on the reduction of liver fat, a marker of insulin resistance and increased cardiovascular risk [11, 12], beyond the established benefits of the traditional MED diet [8]. Furthermore, we suggested that the Green-MED diet might modify the gut microbiome in a manner that optimizes the efficacy of autologous fecal microbiota transplantation for the prevention of weight regain [13].

Only a minor proportion of ingested polyphenols are absorbed in the upper gastrointestinal tract, with estimates ranging between 5 and 10%. The remaining fraction of polyphenols are metabolized by the gut microbiome, with microbial-derived phenolic metabolites subsequently absorbed by the portal vein, ultimately reaching the systemic circulation as bioactive compounds [14, 15]. It was previously shown that polyphenols are not merely subject to microbial metabolism, but that certain doses of selected polyphenols may modify the gut microbial composition, thus forming a bidirectional relationship [14, 15]. For example, flavonoids, the dominant class of polyphenol compounds found in Mankai [16], are metabolized and transformed by the gut microbiome, funneling this diverse range of compounds to a reduced number of metabolites, while altering the taxonomic structure of the gut microbial community [17].

Another group of metabolites shown to be affected by the gut microbiome is circulating branched-chain amino acids (BCAA). It was previously established that dysregulation of BCAA metabolism is a hallmark of obesity and insulin resistance, with obese/insulin-resistant individuals being characterized by elevated levels of serum BCAA [18]. It was later suggested that variation in circulating BCAA levels, and subsequently the host’s metabolic state, are driven by microbial biosynthesis and uptake in the gut microbiome [19].

The previously reported DIRECT-PLUS dietary trial was a three-armed randomized trial including 294 subjects, with primary outcomes that included abdominal fat deposition and obesity [8, 9]. In the current analysis, predefined as a secondary outcome, we evaluated the effect of MED-based dietary interventions on the gut microbiome composition and function. We sought to identify specific microbial genera and metabolic pathways modified by the plant-enriched Green-MED diet while shedding light on the important role of the rare, “non-core” taxa as drivers of change in composition. Lastly, we aimed to determine the mediating role of the gut microbiome in the association between dietary adherence and the notable cardiometabolic risk reduction introduced by the Green-MED diet.

## Methods

### Recruitment to the study

The DIRECT-PLUS (clinicaltrials.gov ID: NCT03020186) was a prospectively registered clinical trial carried out in the Nuclear Research Center Negev, Dimona, Israel, a facility with an in-house dining room and medical clinic. The first subject was enrolled on January 28, 2017, and the last subject was enrolled on April 30, 2017. The trial was initiated and conducted in a single phase between May 2017 and November 2018.

Inclusion criteria were age > 30 years with abdominal obesity [waist circumference (WC): men > 102 cm, women > 88 cm] or dyslipidemia [triglycerides (TG) > 150mg/dL and high-density lipoprotein cholesterol (HDLc) ≤ 40 mg/dL for men, ≤ 50 mg/dL for women]. Exclusion criteria are detailed in Additional file 1: Methods. The study was approved by the Soroka University Medical Centre Institutional Review Board. Participants provided written informed consent and received no compensation.

### Study design, randomization, and intervention

Design and intervention of the DIRECT-PLUS, as well as primary outcomes of the trial (defined as abdominal fat tissues, body weight, and waist circumference), were previously reported in detail [8, 9]. This report is focused on the analysis of the trials’ secondary outcomes, including gut microbiome profile, lipid profile, glycemic control, inflammatory state, and cardiometabolic risk.

All eligible participants were randomized in a 1:1:1 ratio, stratified by gender and work site (to ensure equal workplace-related lifestyle features between groups), into one of the three intervention groups: healthy dietary guidelines (HDG), MED, Green-MED, all combined with physical activity accommodation (Additional file 2: Table S1).

The trial was conducted simultaneously, with all participants randomized and followed in a single phase. Participants were aware of their assigned dietary group (open-label protocol). All participants were provided with a free gym membership and were guided to engage in moderate-intensity physical activity (Additional file 1: Methods).

Dietary guidance was provided as follows, with sessions identical in time and guidance across intervention groups to achieve equivalent intervention intensity: (1) HDG group: In addition to physical activity, participants received standard nutritional counseling based on the Harvard T. H. Chan School of Public Health’s “The Nutrition Source,” a website sharing informed nutrition knowledge with the public, providing science-based guidance for healthy living. (2) MED group: In addition to physical activity, participants were instructed to adopt a calorie-restricted Mediterranean diet [2, 20]. The MED diet assigned was rich in vegetables, with poultry and fish replacing beef and lamb intake. The diet also included 28g/day of walnuts.

(3) Green-MED group: In addition to physical activity and the provision of 28g/day walnuts, the Green-MED diet was further restricted in processed and red meat and was richer in plants and polyphenols. The participants were provided with 3–4 cups/day of green tea and 100g/day of frozen *Wolffia globosa* (Mankai strain) plant frozen cubes, as a green shake for dinner. Both the MED and green-MED diets were equally calorie-restricted (1500–1800kcal/day for men and 1200–1400kcal/day for women). Two hundred eighty-six subjects, all of which provided a fecal sample at baseline (97%), were included in the current analyses.

Details regarding the lifestyle interventions and motivation techniques are provided in Additional file 1: Methods.

### Anthropometric measurements and blood and stool sample collection

Measurements and sample collection were obtained at baseline and 6 months. Participants were weighed without shoes to the nearest 0.1 kg. Waist circumference was measured halfway between the last rib and the iliac crest to the nearest millimeter.

Two blood pressure (BP) measurements and pulse were recorded after resting, using an automatic BP monitor (Accutorr-4; Datascope). Blood pressure was calculated as the mean of the two measurements.

Blood samples were obtained after a 12-h fast, centrifuged, and stored at –80°C pending analyses; further information regarding the assays can be found in Additional file 1: Methods. The 10-year Framingham risk score (FRS) was calculated based on gender, age, total cholesterol and HDLc levels, systolic BP and antihypertensive drug dependence, smoking status, and diabetes [21].

Fecal samples were collected at the study site, immediately frozen to –20°C for 1–3 days, then transferred to –80°C pending DNA extraction. Participants prescribed antibiotic therapy 2 months prior to the delivery of baseline fecal samples were excluded from all microbiome analyses. All blood biochemical assays were performed at the University of Leipzig, Germany. 16S rRNA gene sequencing was performed at Fondazione Edmund Mach, Italy. Fecal metagenomic sequencing was performed at CoreBiome, MN, USA.

### 16S rRNA sequencing and processing

To characterize the microbiome composition and to assess taxon abundance, fecal DNA from 546 fecal samples was extracted and 16S rRNA gene amplicon sequencing was performed by targeting the V3–V4 variable region of approximately 460-bp length using the bacterial primer pair 341F (5′ CCTACGGGNGGCWGCAG 3′) and 806R (5′ GACTACNVGGGTWTCTAATCC 3′). Paired-end sequencing reads were quality filtered, trimmed, de-noised, and merged using the DADA2 software version 1.14.0 [22]. To reject low-quality bases, forward reads were truncated to 280 bases and 25 bases were cropped from start. Reverse reads were truncated to 220 bases and 14 bases were cropped from start. Reads with a number of expected errors higher than two were discarded. Taxonomy was assigned to all identified amplicon sequence variants (ASVs) based on the SILVA rRNA reference database version 132 [23].

### Shotgun metagenomics sequencing and processing

To characterize the microbiome’s functional capacity, a subset of 173 samples (31% of samples) underwent shotgun metagenomics. Samples were collected from 90 participants as part of the pre-transplantation sampling of the DIRECT-PLUS autologous fecal microbiota transplantation study [13], with 31 samples from the HDG group, 69 from the MED group, and 73 from the Green-MED group. DNA was extracted, sequenced, and normalized with an average depth of 15.4±2.6 million reads per sample (mean±standard deviation). DNA sequences were aligned using an accelerated version of the Needleman-Wunsch algorithm to a curated database containing all representative genomes in RefSeq v86 [24]. Each input sequence was assigned the lowest common ancestor that was consistent across at least 80% of all reference sequences tied for best hit. Kyoto Encyclopedia of Genes and Genomes (KEGG) Orthology groups were observed directly using alignment against a gene database derived from the strain database used above. Further details regarding DNA extraction and bioinformatics pipelines can be found in Additional file 1: Methods.

### Dietary and adherence assessment

Assessment of nutritional intake and lifestyle habits was performed using self-reported food frequency questionnaires administered through a computer at baseline and after 6 months [25, 26]. Frequencies and portions of each individual food item were converted to average daily intake for each participant at each time point. Average daily energy intake was calculated by multiplying the frequency of consumption of each item by its caloric content and summing across all foods.

We then applied a Green-MED adherence score to measure the degree of adherence to the Green-MED pattern. The adherence score was created based on the MED index by Willett et al. [27] and Trichopoulou et al. [28] and modified to suit the Green-MED intervention. The adherence score was based on the intake of 9 items: walnuts, vegetables, processed meat, red meat, legumes, fruits, fish, green tea, and Mankai. Each component’s daily intake was normalized to the average daily intake (component intake in grams/total calories). For beneficial components (vegetables, legumes, fruit, walnuts, fish, green tea, and Mankai), individuals whose consumption was below the median were assigned a value of 0, and those whose consumption was at or above the median were assigned a value of 1. For red/processed meat intake, participants whose consumption was below the median were assigned a value of 1, and those at or above the median were assigned a value of 0. The final Green-MED score ranged from 0 (minimal adherence) to 9 (perfect adherence).

### Statistical analysis

All statistical analyses were performed in R v3.5.0. To assess the effect of dietary intervention on the composition of microbial communities, we applied permutational multivariate analysis of variance (PERMANOVA) on dissimilarity matrices of weighted UniFrac, using the adonis function from the R package vegan with 999 permutations [29]. To further evaluate the intervention effect on the microbiome composition over time, a PERMANOVA model was applied with the interaction term of lifestyle intervention and group as independent variables (group X time), both for all groups together and for all three combinations of group pairs in a leave-one-out manner.

To quantify the percentage of variance explained by change in biomarkers and dietary variables on the gut microbiome’s composition, vectors of person-specific ASV changes were calculated for each ASV per individual as follows: ${Log}_2\frac{ASV_{t6}}{ASV_{t0}}$. Euclidian distances between the vectors were calculated, forming a distance matrix between all individuals. Next, a PERMANOVA model was applied with the distance matrix as the dependent variable and percent change of biomarkers or dietary intake score as independent variables. All the *P* values from the PERMANOVA were corrected for multiple comparisons using the Benjamini-Hochberg procedure. For all analyses stratified by core/non-core taxonomic features, the cutoff was defined as >50% prevalence [30]. To assess to what extent the intervention effect on the gut microbiome was convergent by nature, we generated a distance matrix between all subjects at 6 months, measured in weighted UniFrac and based on the 16S rRNA sequences. We next compared the observed differences between the mean within-group and between-group dissimilarities, to a null model generated by 10^6^ permutations of random sample labeling. For per-feature analysis, we used the R package MaAsLin2 [31], with microbial features, taxonomic (16S rRNA) or pathways (shotgun sequencing) filtered, leaving features with a relative abundance of 10^−5^ in at least 5% of samples. To detect differences in changes of microbial features between groups over time, we built linear mixed models that include group, time, and their product term, with participant identifier as random. A significant *P* value for the product term indicates that over time changes in microbial features differ between groups. For changes in biomarkers and specific microbial features, we determined the gradual dietary effect using Kendall’s tau-b correlation on the percent change in each biomarker across dietary groups and used generalized linear models to assess between-group differences. Dietary groups were considered a ranked variable, taking into account the increase in polyphenols and decrease in red and processed meat across the groups. To assess the correlation between specific taxa and biomarkers, the Spearman correlation test was used.

Assessing the mediatory effect of the microbiome, we first employed the meditation analysis suggested by Imai et al. [32] by the “mediation” package in R [33], with the Green-MED score being the independent variable, PCo1 loading of the log2 change matrix accounting for mediation by microbiome composition change, and biomarkers being the dependent variables.

We next used the high-dimensional mediation analysis suggested by Zhang et al. [34], using the HIMA package in R [34], using the same dependent/independent variables, with the change of addressing the high-dimensional nature of the microbiome data, and identifying changes in specific taxa as mediators. In the per-feature tests, unless otherwise noted, all the *P* values were corrected for multiple comparisons using the Benjamini-Hochberg procedure. All multiple hypothesis testing was controlled for false discovery rate with a target of *q* < 0.25.

## Results

### Baseline characteristics of participants and adherence rates in the DIRECT-PLUS trial

Two hundred ninety-four subjects were recruited in the DIRECT-PLUS weight-loss trial [8, 9] and randomized to one of the three lifestyle intervention groups.

**Fig. 1:**
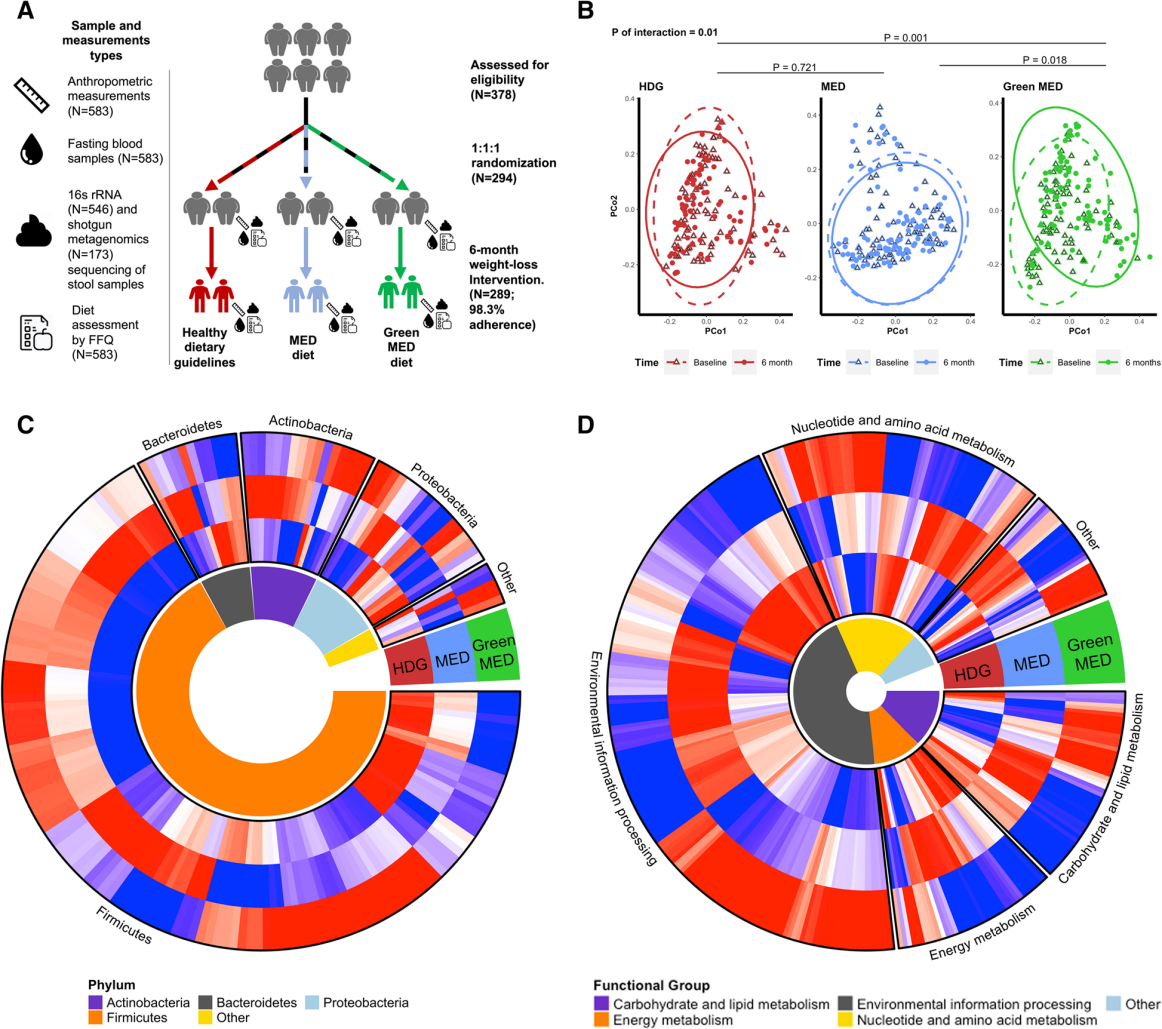
Changes in gut microbiome composition following 6-month dietary interventions. **A** Study design, adherence rate, and total sample count by category. **B** PCoA of all samples by group and time. The Green-MED group had undergone the most prominent compositional shift, with a significant interaction between group and time. *P* values denote the significance level of time-group interaction terms with all three groups (bold) and group-pair combinations by leave-one-out analysis (PERMANOVA). **C**, **D** Heatmap of changes in genus-level bacteria (**C**) and KEGG metabolizing modules (**D**) by lifestyle intervention group. For each cell, colors indicate the within-group change coefficient by MaAsLin2 between baseline and 6 months. The three dietary arms lead to distinct changes in patterns across genera and microbial metabolic pathways

Of those, 97% (*n* = 286) provided a fecal sample at baseline and were included in the current analyses. We measured body weight, waist circumference, and blood pressure and collected fasting blood samples, stool samples, and dietary data at baseline and month 6. Of the 546 stool samples profiled by 16S rRNA gene sequencing, 173 were further profiled by shotgun metagenomic sequencing [13]. The mean age at baseline was 51 years; 88% were male, with a mean body mass index (BMI) of 31 kg/m^2^. Follow-up stool samples were obtained from 91% of the recruited participants. See Additional file 3: Table S2 for group characteristics, Fig. 1A for the study design, Additional file 4: Fig. S1 for the flow chart, and Additional file 4: Fig. S2 for patterns of gut microbial taxonomic variation of the cohort.

### The Green-MED diet modifies the composition and function of the gut microbiome

Following 6 months of dieting, all three groups had undergone a significant change in microbiome composition, as assessed by 16S rRNA sequencing, though it appeared that the Green-MED dieters exhibited the most prominent change, with a significant interaction between time (baseline to six months) and group (*P* of interaction = 0.01) as revealed by principal coordinates analysis (PCoA) based on weighted UniFrac distances. To compare the compositional change between groups in a subject-specific manner, we created a log2 change ASV matrix between the two timepoints of each subject. The Green-MED group differed significantly in its change compared to the other two groups (*P*_Green-MED vs HDG_ = 0.001, *P*_Green-MED vs MED_ = 0.04), whereas no significant difference was observed between the MED and HDG groups (*P*_MED vs HDG_ = 0.19; Fig. 1B and Additional file 4: Fig. S3). We next evaluated the effect of diet on change at the genus level of bacteria and KEGG pathway modules. At month 6, different dietary interventions led to distinct changes across different phyla and functional groups (Fig. 1C, D).

### The non-core microbiome as a driver of general composition change

**Fig. 2:**
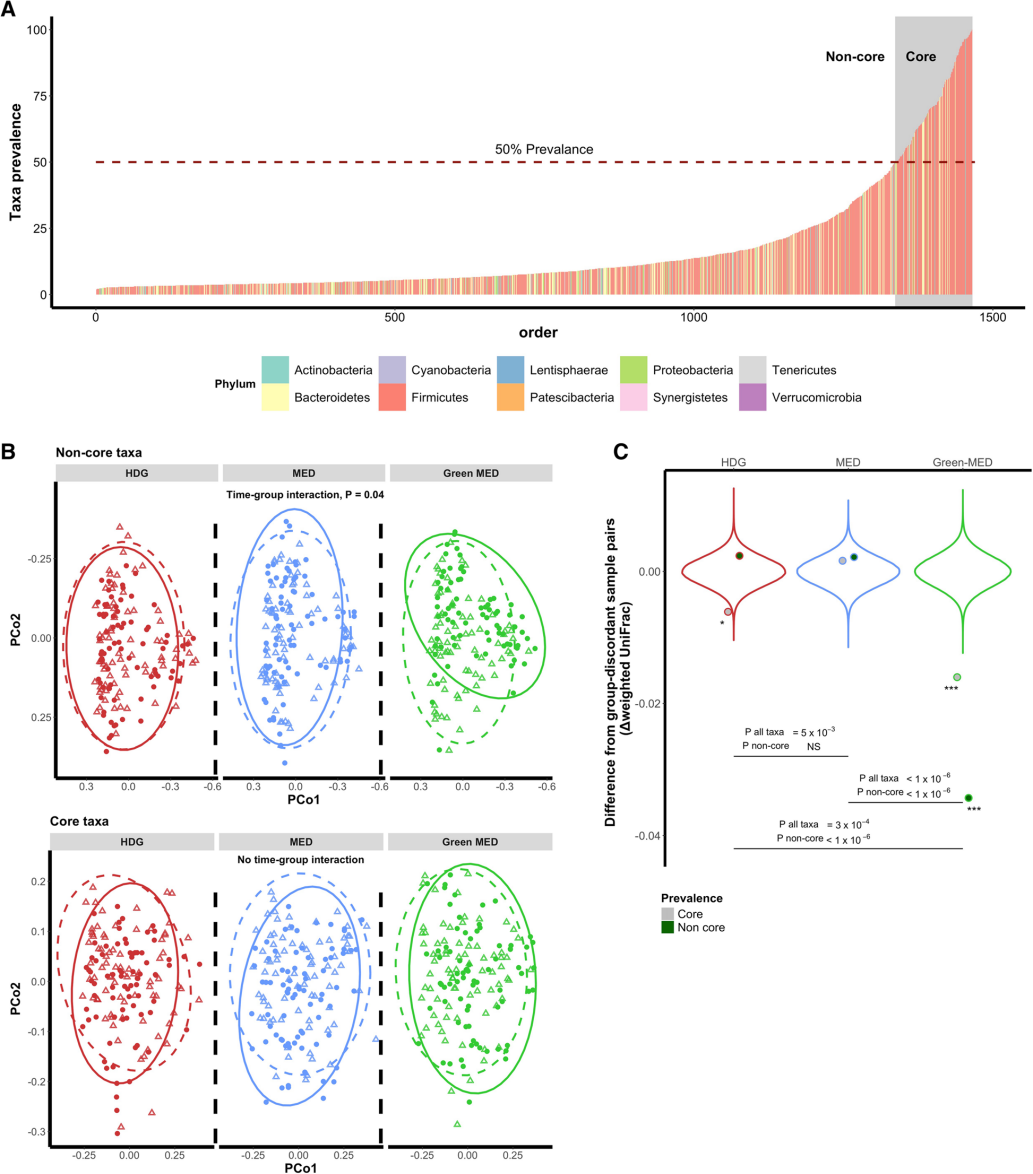
The non-core microbiome as a driver of general composition change. **A** All ASVs with prevalence above 3%, ordered by prevalence across all samples. “core” taxa were defined as > 50% prevalence, with the remaining taxa defined as “non-core.” **B** PCoA stratified by the non-core (<50% prevalence, top) and core microbiome (>50% prevalence, bottom). The compositional shift of the Green-MED group originated from the non-core (rare) taxa with a significant interaction between time and group in PERMANOVA. **C** Differences in within-group dissimilarity and between-group dissimilarity, by non-core (green points) and all taxa (gray points) microbiome composition. The Green-MED dieters’ microbiome composition assimilated each other at the 6-month point. This effect was even more dominant in the non-core fraction of the microbiome. Violin plots describe the distribution of 10^6^ random permutations of the same measure, shuffling sample labels at each iteration

In light of evidence linking the rare, person-specific, fraction of taxa in the microbiome profile, with sensitivity to dietary alterations [35], we next stratified the taxonomic features, as assessed by 16S rRNA sequencing, into “core” (ASVs > 50% prevalence) and “non-core” categories (ASVs < 50% prevalence). We found that the changes in taxonomy induced by the Green-MED were largely driven by the non-core features (Fig. 2A, B).

We next asked the question whether the dietary regimens modified the composition of the microbiome in a converging manner, i.e., whether changes in the microbiome compositions of participants resulted in the subject’s gut microbiomes being more similar to each other during the intervention, and to what extent the converging effect was affected by the dietary regimen. Comparing within-group and between-group dissimilarities to a null model, we observed that the Green-MED dieters’ microbiome composition became more similar following the diet, as compared to the HDG and MED groups. Furthermore, performing the same comparison only on the non-core taxonomic features, it could be observed that convergence was more dominant in the non-core microbiome (Fig. 2C).

### The Green-MED diet increases abundance of *Prevotella* and BCAA degrading pathways and decreases *Bifidobacterium* and BCAA biosynthesis pathways in the gut microbiome

**Fig. 3:**
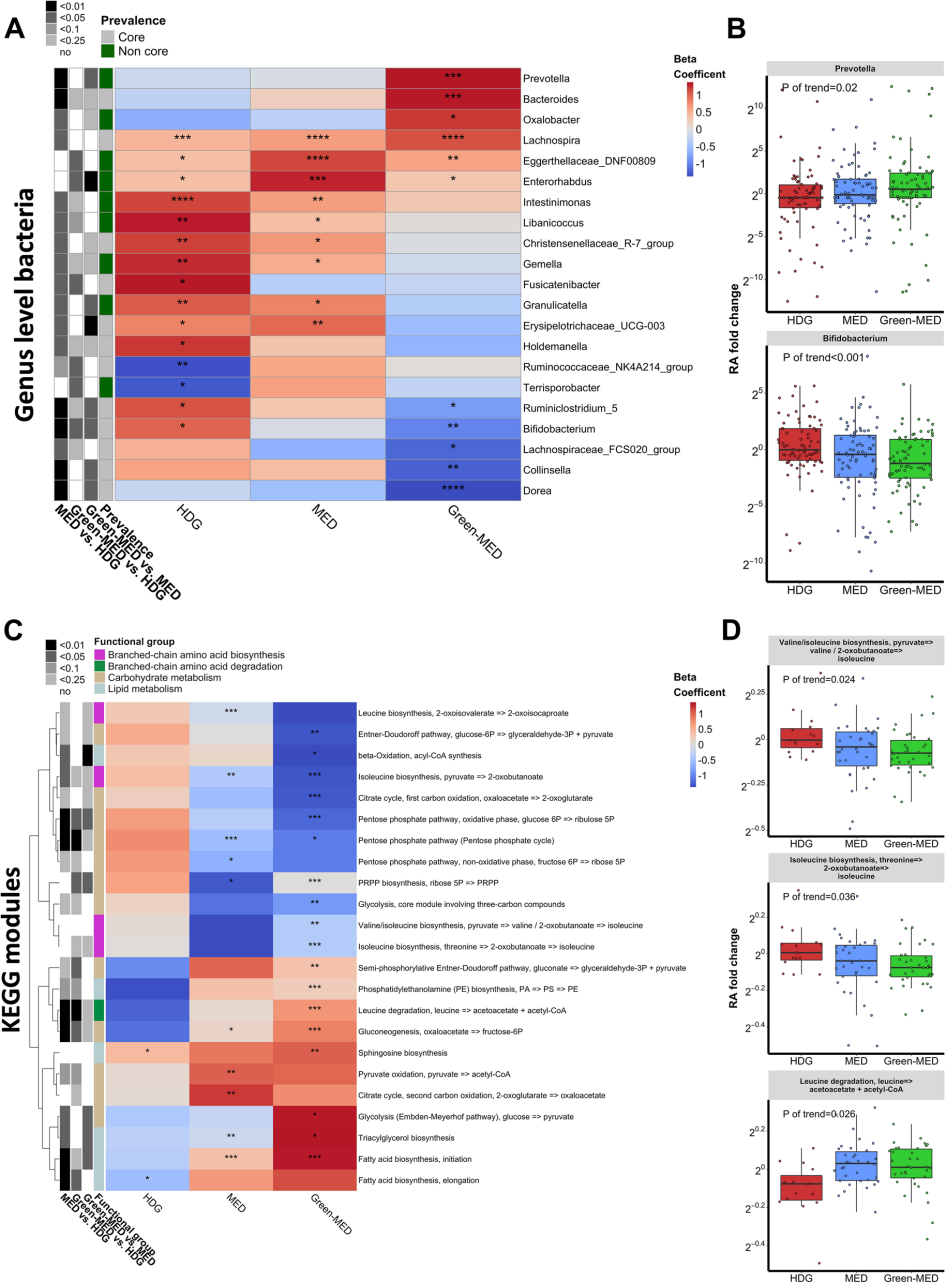
MED diets’ effects on microbial genera and metabolic pathways. **A** Heatmap of changes in genus-level bacteria by lifestyle intervention group, for taxa with significant time*group interaction. For each cell, colors indicate the within-group change coefficient over time and asterisks denote significance. Black-White annotations on the left denote the significance of between-group change difference (by MaAsLin2 time*group interaction). **B** Green-MED was distinguished by several changes, including an increase in *Prevotella* abundance and a decrease in *Bifidobacterium.* **C** Heatmap of changes in metabolism-related KEGG modules by lifestyle intervention group, for pathways with significant time*group interaction. For each cell, colors indicate the within-group change coefficient over time and asterisks denote significance. Black-white annotations on the left denote the significance of between-group change difference (by MaAsLin2 time*group interaction). Colors on the left denote the metabolism category. **D** The Green-MED diet was associated with increased BCAA degradation pathways and decreased BCAA biosynthesis*.* ****FDR < 0.001, ***FDR < 0.01, **FDR < 0.05, *FDR < 0.25

We conducted per-feature testing by using MaAsLin2 linear mixed models [31] to identify microbial genera (by 16S rRNA) and KEGG modules (by shotgun metagenomics) that were differentially changed by diet from baseline to month 6. We found that 21 genera responded to the dietary intervention differentially across groups (*q* of interaction < 0.25; Fig. 3A), including a stepwise increase in *Prevotella* across the three dietary groups, suggesting an effect induced by the graded dietary modifications across groups (Kendall tau; *P* = 0.02), and a stepwise decrease in *Bifidobacterium* across the groups (Kendall tau; *P* < 0.001). These observations are in accordance with previous reports that linked vegetable-based diets [4] and adherence to a MED diet with increased *Prevotella* abundance [5] and linked greater weight loss [36] and a dietary pattern with improved glycemic index to lower abundance of *Bifidobacterium* spp. [37].

Comparing changes in microbial metabolic pathways (KEGG modules) that utilize dietary components (i.e., carbohydrate, lipid, amino acid metabolism), we found a stepwise increase in BCAA degrading pathways (isoleucine degradation, Kendall tau; *P* = 0.02) and a decrease in pathways of BCAA biosynthesis (valine and isoleucine biosynthesis, Kendall tau; *P* < 0.05) comparing MED diet groups to the HDG group. MED diet interventions also led to an enrichment in lipid metabolizing pathways, possibly due to the increased lipid intake and decreased carbohydrate intake commensurate with MED diets (Fig. 3C, D).

### The beneficial effects of MED diets on cardiometabolic risk factors were attributable to plant-sourced components

**Fig. 4:**
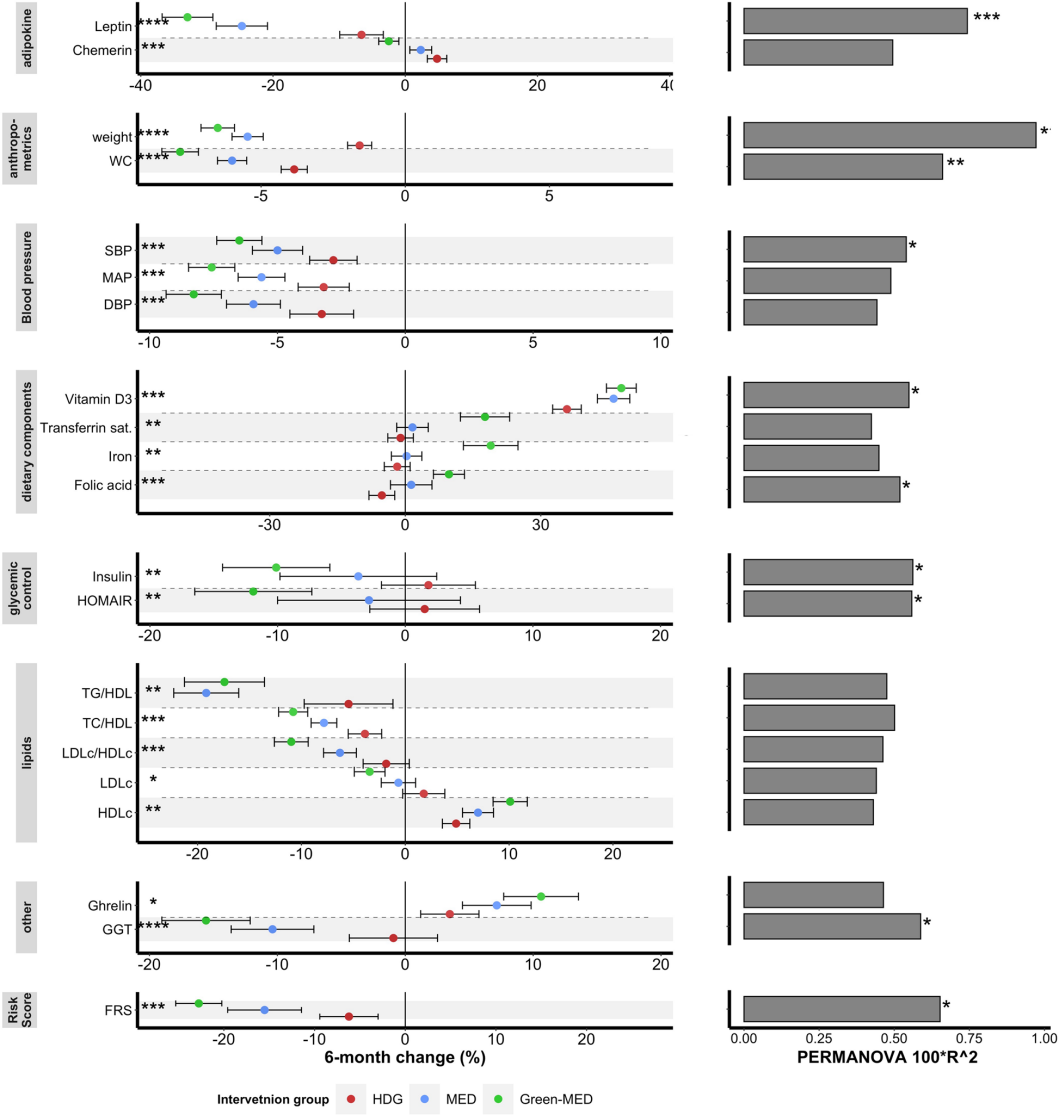
Six-month changes in cardiometabolic markers and their associations with changes in global microbiome composition. Left: Forest plot of percent change in cardiometabolic health markers following 6 months of dietary intervention across groups. Significant changes in biomarkers are shown (FDR-corrected *P* Kendell tau < 0.25). Right: Associations between changes in the corresponding biomarker on the left and composition change (log2 fold change of all ASVs) by performing PERMANOVA test on a Euclidean distance matrix. The Green-MED diet improved cardiometabolic health, with changes in a subset of biomarkers, including body weight, waist circumference, blood pressure, and glycemic profile, being associated with microbiome compositional shift. ****FDR < 0.001, ***FDR < 0.01, **FDR < 0.05, *FDR < 0.25. FRS, Framingham Risk Score; GGT, gamma glutamyl transferase; SBP/DBP, systolic/diastolic blood pressure; MAP, mean arterial pressure; HDG, healthy dietary guidance; HDLc, high-density lipoprotein cholesterol; HOMA-IR, homoeostatic model assessment of insulin resistance; LDLc, low-density lipoprotein cholesterol; MED, Mediterranean; TC, total cholesterol; TG, triglycerides

To examine the effects of plant-enriched and meat-depleted diets on cardiometabolic markers, we evaluated the 6-month change in a graded manner across the dietary groups. Among 47 blood biomarkers and anthropometric measurements, significant effects of the dietary intervention were found in 21 (Kendall tau, FDR-corrected *q* < 0.25). As we recently reported, the Green-MED diet led to the greatest body-weight reduction (−6.5%), followed by MED (−5.4%) and the HDG group (−1.58%, *q* < 0.001) [8]. Similar cross-group trends were observed in the reduction of Framingham risk score, waist circumference, mean arterial pressure, and homoeostatic model assessment of insulin resistance (HOMA-IR) (Fig. 4), along with diet-related markers of iron, vitamin D3, and folic acid, the latter being of interest as an established biomarker of green leaf consumption [38].

Evaluating the association between changes in biomarkers and shifts in microbiome composition (assessed by 16S rRNA sequencing), body-weight change explained the highest proportion of variation (0.9%). Significant associations with compositional changes were observed in the change in several biomarkers, including plasma leptin (0.7%), waist circumference (0.66%), systolic blood pressure (0.5%), HOMA-IR (0.5%), and Framingham risk score (0.65%, Fig. 4).

### Changes in gut microbiome mediate the protective effect of adherence to the Green-MED diet on cardiometabolic risk factors

**Fig. 5:**
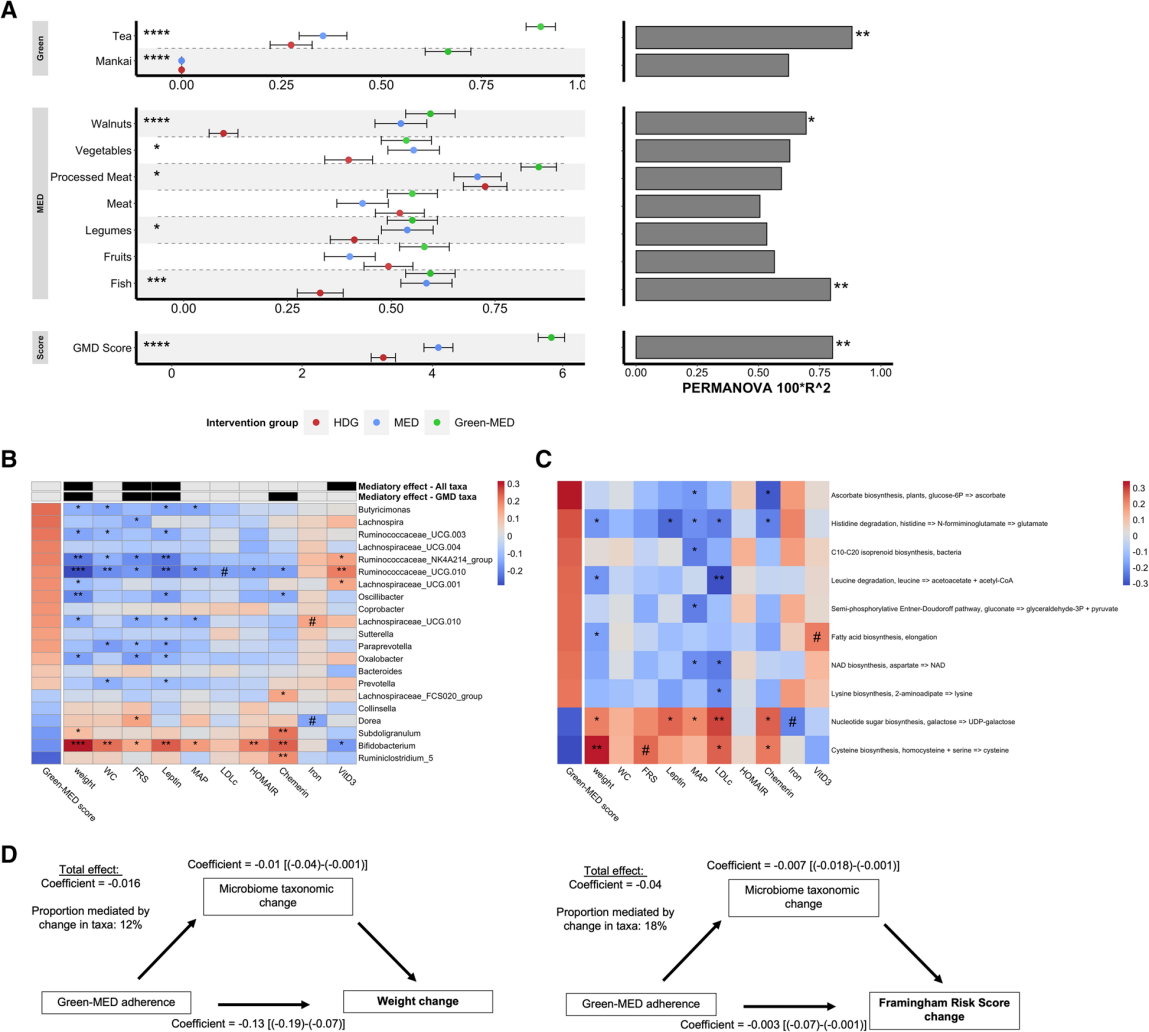
Green-MED adherence, cardiometabolic markers, and the association with specific bacteria and microbial metabolic pathways. **A** Left: Forest plot of Green-MED adherence score and its components following 6 months of dietary intervention across groups. Adherence was assessed using a 9-dimensional index, ranging from 0 (non-adherence) to 9 (full adherence). Right: Associations between changes in the corresponding dietary component on the left and compositional change (log2 fold change of all ASVs) by performing PERMANOVA test on the Euclidean distance matrix. Green-MED dieters adhered to their assigned intervention, with intake of specific components (tea and walnuts) being associated with microbiome compositional shift. **B**, **C** Heatmap showing the association patterns of changes in the Green-MED adherence score and change in genus taxa (**B**) and KEGG modules (**C**). Features are arranged from top to bottom by their correlations with the adherence score. For each cell, colors indicate the Spearman rho values. Several links between change in microbial features and change in biomarkers were observed. Specifically, changes in specific Green-MED-associated bacteria and microbial metabolic, e.g., *Bifidobacterium* and Leucin (BCAA) degradation, were associated with weight change following the intervention. **D** Mediation analysis: assessing the proportional mediatory effect of change in all genus-level taxa (calculated as #1 principal component of their change matrix) in the association between lifestyle intervention and weight change. Weight loss and Framingham risk score were found to be partially mediated by changes in the gut microbiome. ****FDR < 0.001, ***FDR < 0.01, **FDR < 0.05, *FDR < 0.25, ^#^*P* value < 0.05. FRS, Framingham risk score; MAP, mean arterial pressure; HDG, healthy dietary guidance; HOMA-IR, homoeostatic model assessment of insulin resistance; LDLc, low-density lipoprotein cholesterol; MED, Mediterranean

In order to assess the interplay between adherence to the Green-MED diet, gut microbial changes, and cardiometabolic effects, we devised a 9-dimensional adherence score (the “Methods” section), modified from the original Mediterranean index [27, 39, 40], with the addition of green components (green tea and Mankai) and the reductions in both red and processed meat intake. The green-MED score ranged from 0 (non-adherence) to 9 (full adherence, Fig. 5A) and was standardized based on caloric intake. As expected, the Green-MED dieters had higher adherence to green tea and Mankai intake and reduced intake of processed meat. Both MED groups adhered to increased intake of vegetables, legumes, and walnuts. Overall, the Green-MED score had a significant positive increment across dietary groups (MED vs. HDG *P* = 0.03, Green-MED vs. HDG *P* < 0.001; Kendall tau, *P* < 0.001; Fig. 5A). Furthermore, the Green-MED score was significantly associated with gut microbiome compositional changes (16S rRNA PERMANOVA, 0.8% explained variance, *P* = 0.006).

We next identified 21 specific genus-level features (by 16S rRNA sequencing) associated with the Green-MED diet, i.e., features with 6-month changes in abundance significantly associated with the Green-MED adherence score or significantly different across the groups (FDR-corrected *q* < 0.25). Among Green-MED-associated taxonomic features, we found that change in *Prevotella* abundance was positively associated with the Green-MED adherence score, while *Bifidobacterium* was inversely associated with the score (Fig. 5B).

Following the identification of Green-MED-associated bacteria, we examined the association of the specific taxonomic features with cardiometabolic risk markers and found that a reduction in *Bifidobacterium* abundance was significantly associated with weight loss (Spearman’s rho = 0.31, FDR-corrected *q* < 0.001). We also found that increased abundance of several genera from the *Ruminococcaceae* family was associated with weight loss, namely *Ruminococcaceae UCG10* (Spearman’s rho = −0.28, FDR-corrected *q* < 0.001), *Ruminococcaceae NK4A214 group* (rho = −0.21, FDR-corrected *q* = 0.005), and *Ruminococcaceae UCG3* (rho = −0.14, FDR-corrected *q* = 0.08). In addition, a reduction in *Bifidobacterium* and *Dorea* and an increase in genera from the *Ruminococcaceae* and *Lachnospiraceae* families were associated with a reduction in Framingham risk score (Fig. 5B).

In a similar analysis performed on KEGG modules (assessed by shotgun sequencing), we identified significant associations of the Green-MED adherence score with 10 microbial metabolic pathways (FDR-corrected *q* < 0.25). The leucine degradation pathway was positively correlated with Green-MED adherence (Spearman’s rho = −0.21, FDR-corrected *q* = 0.165). Reduction in the levels of the cysteine biosynthesis module was associated with reductions of body weight (Spearman’s rho = 0.33, FDR-corrected *q* = 0.02) and Framingham risk score (Spearman’s rho = 0.25, *P* = 0.03), an observation that is consistent with a previous report demonstrating that high abundance of cysteine biosynthesis pathways is associated with unsuccessful weight loss [41] (Fig. 5C).

As we hypothesized that the dietary interventions, through modulation of the gut microbiome, exert their effects on cardiometabolic risk, we next sought to quantify the mediation effect. To this aim, we estimated the causal mediation effect as proposed by Imai et al. [32], using the first principal component of variation of the ASV change matrix as a summary measure of the gut microbiome composition. The association between the Green-MED diet adherence and weight loss (12% mediation by taxonomic change), WC loss (10%), and FRS reduction (18%) was found to be mediated by the change in the gut microbial composition. When assessing the mediatory effect specific to the change of Green-MED-associated genera, Chemerin change was also found to be significantly mediated (Fig. 5B, D). In order to assess the mediatory effect of specific bacteria, we applied a high-dimensional mediation analysis [34]. Among the significant mediatory taxa, *Bifidobacterium* change was found to mediate the Green-MED effect on weight loss (4%) and *Ruminococcaceae UCG10* mediated the effect on FRS reduction (1.3%). Changes in the cysteine biosynthesis pathway were a prominent mediator of weight loss (14.2%) and FRS reduction (29.3%).

## Discussion

In the current study, we demonstrate the effect of MED and Green-MED diets on the gut microbiome and determine the mediatory role of the microbiome in the associations between adherence to the Green-MED diet and subsequent improvement in markers of cardiometabolic risk. We showed that the Green-MED diet induced a prominent change in the gut microbiome composition, driven by the low-prevalent “non-core” fraction of the gut microbiome. This change was convergent by nature, with the taxonomic composition of the microbiome of individuals in the Green-MED becoming more similar to each other. The different diets were also characterized by distinct taxonomic and functional changes, inducing a gradual increase in the abundance of *Prevotella* and BCAA degradation pathway concomitant with the increment of plant components in MED diets, and reductions in the abundance of *Bifidobacterium* and BCAA biosynthesis pathways when comparing MED diets vs. the control diet. The MED and Green-MED diets improved cardiometabolic markers, including the Framingham risk score, body weight, blood pressure, HOMA-IR, and fasting plasma leptin. These beneficial changes in levels of cardiometabolic biomarkers were associated with a concurrent shift in the gut microbiome composition, estimated to mediate 12% of body-weight reduction and 18% of Framingham risk score reduction.

Our study has several limitations. The high proportion of male participants in the trial (88%) may limit the generalizability of our results to females. However, a previous report showed that, in western populations, gut microbiome composition is not associated with gender [42]. We addressed this limitation by employing gender stratification at the trial’s randomization. Furthermore, assessments of dietary intake and adherence to intervention regimes were based on self-reports. This limitation was addressed by the measurement of serum folic acid, a biomarker reflecting green leaf consumption [38], that increased across groups, by the use of previously validated dietary questionnaires [26, 43] and by focusing the analysis on individual-level dietary changes. As only a subset of approximately one-third of the fecal samples were sequenced by shotgun metagenomics, with a relatively shallow coverage of 15 million reads per sample on average, this study was possibly underpowered to detect subtle functional differences between the gut microbiome of the three intervention groups.

The strengths of the study include the intense dietary instruction and multidisciplinary supervision, the simultaneous design, and the high retention rate.

Although the function and composition of the gut microbiome have dynamic features, with some changes observed on a daily scale, most of this variation is person-specific [44]. Moreover, while short-term dietary changes induce rapid and brief changes in microbiome composition, they tend to regress to the baseline personal microbiome structure [45]. Long-term dietary habits however are considered a major determinant of gut microbiome composition, possibly due to consistent selective pressure induced by the diet and specific nutrients [46, 47]. A recent 1-year dietary trial reported that the MED diet showed a notable impact on the composition of the gut microbiome [48]. In the current analysis, we showed that long-term adherence to the Green-MED diet prompted a greater composition change than the shift induced by the traditional MED diet. Furthermore, we demonstrated that the Green-MED composition change was convergent by nature (i.e., the microbiome of the Green-MED dieters became more similar to each other), corroborating the hypothesis that change was driven by a selective pressure [49]. Interestingly enough, convergence was likely driven by the rare, non-core fraction of the microbiome, presumably utilizing its pool of genetic resources given a change in environmental conditions [50].

The major determinants differentiating the Green-MED diet from the MED diet are the supplementation of Mankai and green tea intake while reducing the consumption of red and processed meat.

Mankai was previously established as a high-quality substitute source for animal protein in a controlled test meal, with blood concentrations of essential amino acids being similar after Mankai intake to that of well-established animal and plant protein sources. As an exception, Mankai intake induced only a minor increase in circulating BCAA levels [51]. This observation is in accord with our current analysis, showing that the Green-MED diet was associated with a reduction in the gut microbiomes’ capacity for BCAA biosynthesis and an increase in BCAA degradation pathways.

Furthermore, both Mankai and green tea were shown to be rich sources of polyphenols [16, 52, 53], with Mankai containing ~200 different phenolic metabolites. We previously reported that in a Mankai-supplemented artificial gut bioreactor, the functional profile of the gut microbiome shifted towards polyphenol degradation, e.g., degradation of the phenolic compounds protocatechuate and catechol [52]. Of note, in this study, we were not able to identify a differential effect of the Green-MED diet on polyphenol-related pathways, possibly due to our power limitation mentioned above.

While the Mankai plant is considered fiber-rich, with a fiber content of ~25% [54], the Green-MED diet lead to an increase in *Prevotella* abundance, a genus previously associated with high fiber intake [55] and vegetarian diets [47, 56]. Moreover, low abundance of *Prevotella* was shown to be a hallmark for the transition to a western lifestyle [56]. Previous studies have reached contradictory conclusions regarding the role of the dominant *Prevotella* species, *Prevotella copri*, in host metabolism. It was recently suggested that the abundance of *Prevotella copri*, as an indication of the preexisting, diet-related biosynthetic capacity of the gut microbiome, potentially attenuates the protective effect of the Mediterranean diet on cardiometabolic disease risk [5]. Furthermore, De Vadder et al. demonstrated that elevated *Prevotella copri* abundance is associated with improvements in glucose metabolism and insulin sensitivity by the production of succinate [57], while a study by Pedersen et al. suggested that *Prevotella copri* abundance is associated with insulin resistance, presumably due to increased BCAA biosynthesis [19]. Here, we report that across MED diets with a gradual increment in plant components, intervention intensity was associated with a reduction in BCAA biosynthesis, an increase in BCAA degradation pathways, and increased insulin sensitivity, measured as HOMA-IR. These findings support the hypothesis that *Prevotella* may not be beneficial or deleterious per se, but should be examined in light of other environmental and host-specific factors, requiring further investigations [5, 58]. The association of *Bifidobacterium* with cardiometabolic disease risk has also been inconsistent in previous studies, with an increase in *Bifidobacterium* abundance linked to both successful [59] and unsuccessful [36] weight loss. Our study demonstrated that in the setting of a plant-enriched (green) MED diet, weight loss is associated with a reduction in *Bifidobacterium*.

The associations between dietary patterns, the gut microbiome, and cardiometabolic health have been extensively evaluated in recent years [37, 60, 61], with several studies exploring microbial modifications related to beneficial diets, including the MED diet [4, 5, 48]. Our study links MED diets with several taxa, including the depletion of *Blautia* [48] and enrichment of *Bacteroides* [48], adding to the body of evidence by examining the effect of expanding the plant fraction of the diet while minimizing meat intake. We showed that overall, the gut microbiome contributes a small but significant mediatory effect in the association between MED diets and weight and cardiometabolic risk reduction.

## Conclusions

MED diet enriched in plant components and with decreased red and processed meat consumption (Green-MED diet) has extensive effects on the composition and function of the host gut microbiome, with the latter partially mediating the beneficial effects of the diet on cardiometabolic health. In future studies, the diet-microbiome-host interplay should be further explored and could guide novel beneficial modifications in known dietary patterns.

## Supplementary Information

**Additional file 1.** Methods – Exclusion criteria, Physical activity protocol, Lifestyle sessions and motivation techniques, Blood sample analysis, 16s rRNA sequencing pipeline, Metagenomics analysis BoosterShot pipeline.

**Additional file 2: Table S1**. Outline of the lifestyle interventions.

**Additional file 3: Table S2**. Characteristics of the study population.

**Additional file 4: Figure S1**. Flow Diagram of the DIRECT-PLUS trial.

**Additional file 5.** Contains Figures S2 and S3.

## Data Availability

The 16S rRNA gene and shotgun metagenomic sequencing data have been deposited in the European Nucleotide Archive with accession number PRJEB49146 (16S rRNA sequences; https://www.ebi.ac.uk/ena/browser/view/PRJEB49146) [62] and PRJEB49147 (shotgun sequences; https://www.ebi.ac.uk/ena/browser/view/PRJEB49147) [63].

## Abbreviations

MED: Mediterranean
HDG: Healthy dietary guidelines
BMI: Body mass index
LDLc: Low-density lipoprotein cholesterol
WC: Waist circumference
TG: Triglycerides
HDLc: High-density lipoprotein cholesterol
HOMA-IR: Homoeostatic model assessment of insulin resistance
BP: Blood pressure
FRS: Framingham risk score
BCAA: Branched-chain amino acid
KEGG: Kyoto Encyclopedia of Genes and Genomes
PERMANOVA: Permutational multivariate analysis of variance
PCoA: Principal coordinates analysis
ASV: Amplicon sequence variants

## References

[1] Arnett DK, Blumenthal RS, Albert MA, et al. ACC/AHA guideline on the primary prevention of cardiovascular disease: a report of the American College of Cardiology/American Heart Association Task Force on clinical practice guidelines. Circulation. 2019, 2019;140(11). 10.1161/CIR.0000000000000678 PMC7734661 30879355

[2] Shai I, Schwarzfuchs D, Henkin Y (2008). Weight loss with a low-carbohydrate, Mediterranean, or low-fat diet. N Engl J Med.

[3] American Diabetes Association AD (2018). 4. Lifestyle management: standards of medical care in diabetes-2018. Diabetes Care.

[4] De Filippis F, Pellegrini N, Vannini L (2016). High-level adherence to a Mediterranean diet beneficially impacts the gut microbiota and associated metabolome. Gut.

[5] Wang DD, Nguyen LH, Li Y, et al. The gut microbiome modulates the protective association between a Mediterranean diet and cardiometabolic disease risk. Nat Med. 2021. 10.1038/s41591-020-01223-3 PMC8186452 33574608

[6] Meslier V, Laiola M, Munch H (2020). Gut microbiota Mediterranean diet intervention in overweight and obese subjects lowers plasma cholesterol and causes changes in the gut microbiome and metabolome independently of energy intake. Gut.

[7] Estruch R, Ros E, Salas-Salvadó J (2013). Primary prevention of cardiovascular disease with a Mediterranean diet. N Engl J Med.

[8] Tsaban G, Meir AY, Rinott E (2020). The effect of green Mediterranean diet on cardiometabolic risk; a randomised controlled trial Cardiac risk factors and prevention. Heart.

[9] Yaskolka Meir A, Rinott E, Tsaban G, et al. Effect of green-Mediterranean diet on intrahepatic fat: the DIRECT PLUS randomised controlled trial. Gut. 2020:1–11. 10.1136/gutjnl-2020-323106 PMC8515100 33461965

[10] Huang J, Wang Y, Xie Z, Zhou Y, Zhang Y, Wan X (2014). The anti-obesity effects of green tea in human intervention and basic molecular studies. Eur J Clin Nutr.

[11] Van Herpen NA, Schrauwen-Hinderling VB (2008). Lipid accumulation in non-adipose tissue and lipotoxicity. Physiol Behav.

[12] Byrne CD, Targher G (2015). NAFLD: a multisystem disease. J Hepatol.

[13] Rinott E, Youngster I, Meir AY (2021). Effects of diet-modulated autologous fecal microbiota transplantation on weight regain. Gastroenterology.

[14] Cardona F, Andrés-Lacueva C, Tulipani S, Tinahones FJ, Queipo-Ortuño MI (2013). Benefits of polyphenols on gut microbiota and implications in human health. J Nutr Biochem.

[15] van Duynhoven J, Vaughan EE, Jacobs DM (2011). Metabolic fate of polyphenols in the human superorganism. Proc Natl Acad Sci.

[16] Diotallevi C, Gaudioso G, Fava F (2021). Measuring the effect of Mankai® (Wolffia globosa) on the gut microbiota and its metabolic output using an in vitro colon model. J Funct Foods.

[17] Cassidy A, Minihane A-M (2017). The role of metabolism (and the microbiome) in defining the clinical efficacy of dietary flavonoids. Am J Clin Nutr.

[18] Newgard CB, An J, Bain JR (2009). A branched-chain amino acid-related metabolic signature that differentiates obese and lean humans and contributes to insulin resistance. Cell Metab.

[19] Pedersen HK, Gudmundsdottir V, Nielsen HB (2016). Human gut microbes impact host serum metabolome and insulin sensitivity. Nature.

[20] Gepner Y, Shelef I, Schwarzfuchs D (2018). Effect of distinct lifestyle interventions on mobilization of fat storage pools. Circulation.

[21] D’Agostino RB, Vasan RS, Pencina MJ (2008). General cardiovascular risk profile for use in primary care: the Framingham Heart Study. Circulation.

[22] Callahan BJ, Mcmurdie PJ, Rosen MJ, Han AW, Johnson AJA, Holmes SP. dada2: high-resolution sample inference from illumina amplicon data. 2016;13(7). 10.1038/nmeth.3869 PMC4927377 27214047

[23] Quast C, Pruesse E, Yilmaz P (2013). The SILVA ribosomal RNA gene database project: improved data processing and web-based tools. Nucleic Acids Res.

[24] O’Leary NA, Wright MW, Brister JR (2016). Reference sequence (RefSeq) database at NCBI: current status, taxonomic expansion, and functional annotation. Nucleic Acids Res.

[25] Shai I, Shahar DR, Vardi H, Fraser D (2004). Selection of food items for inclusion in a newly developed food-frequency questionnaire. Public Health Nutr.

[26] Shai I, Rosner BA, Shahar DR (2005). Dietary evaluation and attenuation of relative risk: multiple comparisons between blood and urinary biomarkers, food frequency, and 24-hour recall questionnaires: the DEARR Study. J Nutr.

[27] Willett WC, FS, AT, et al. Mediterranean diet pyramid: a cultural model for healthy eating. Am J Clin Nutr. 1995;61(6 Suppl). 10.1093/ajcn/61.6.1402S 7754995

[28] Trichopoulou A, Costacou T, Bamia C, Trichopoulos D (2003). Adherence to a Mediterranean diet and survival in a Greek population. N Engl J Med.

[29] Oksanen J, Blanchet FG, Kindt R, O’ B, Maintainer H. Vegan: community ecology package [Internet]. 2019.

[30] Wallace RJ, Sasson G, Garnsworthy PC (2019). A heritable subset of the core rumen microbiome dictates dairy cow productivity and emissions. Sci Adv.

[31] Mallick H, Rahnavard A, McIver LJ, et al. Multivariable association discovery in population-scale meta-omics studies. bioRxiv. 2021; 2021.01.20.427420. 10.1371/journal.pcbi.1009442 PMC8714082 34784344

[32] Imai K, Tingley D, Yamamoto T (2013). Experimental designs for identifying causal mechanisms. J R Stat Soc Ser A Stat Soc.

[33] Tingley D, Yamamoto T, Hirose K, Keele L, Imai K (2014). mediation: R package for causal mediation analysis. J Stat Softw.

[34] Zhang H, Zheng Y, Zhang Z (2016). Estimating and testing high-dimensional mediation effects in epigenetic studies. Bioinformatics.

[35] Benjamino J, Lincoln S, Srivastava R, Graf J (2018). Low-abundant bacteria drive compositional changes in the gut microbiota after dietary alteration. Microbiome.

[36] Santacruz A, Marcos A, Wärnberg J (2009). Interplay between weight loss and gut microbiota composition in overweight adolescents. Obesity.

[37] Zeevi D, Korem T, Zmora N (2015). Personalized nutrition by prediction of glycemic responses. Cell.

[38] Moll R, Davis B (2017). Iron, vitamin B12 and folate. Medicine (Baltimore).

[39] Fung TT, McCullough ML, Newby P (2005). Diet-quality scores and plasma concentrations of markers of inflammation and endothelial dysfunction. Am J Clin Nutr.

[40] Estruch R, ER, JS-S, et al. Primary prevention of cardiovascular disease with a Mediterranean diet supplemented with extra-virgin olive oil or nuts. N Engl J Med. 2018;378(25). 10.1056/NEJMoa1800389 29897866

[41] Louis S, Tappu R-M, Damms-Machado A, Huson DH, Bischoff SC (2016). Characterization of the gut microbial community of obese patients following a weight-loss intervention using whole metagenome shotgun sequencing. PLoS One.

[42] Lloyd-Price J, Mahurkar A, Rahnavard G (2017). Strains, functions and dynamics in the expanded Human Microbiome Project. Nature.

[43] Golan R, Schwarzfuchs D, Stampfer MJ, Shai I (2010). DIRECT group. Halo effect of a weight-loss trial on spouses: the DIRECT-Spouse study. Public Health Nutr.

[44] Johnson AJ, Vangay P, Al-Ghalith GA (2019). Daily sampling reveals personalized diet-microbiome associations in humans. Cell Host Microbe.

[45] David LA, Maurice CF, Carmody RN (2014). Diet rapidly and reproducibly alters the human gut microbiome. Nature.

[46] Rothschild D, Weissbrod O, Barkan E (2018). Environment dominates over host genetics in shaping human gut microbiota. Nature.

[47] Wu GD, Chen J, Hoffmann C (2011). Linking long-term dietary patterns with gut microbial enterotypes. Science (80- ).

[48] Shankar Ghosh T, Simone R, Jeffery Ian B (2020). Gut microbiota Mediterranean diet intervention alters the gut microbiome in older people reducing frailty and improving health status: the NU-AGE 1-year dietary intervention across five European countries. Gut.

[49] Foster KR, Schluter J, Coyte KZ, Rakoff-Nahoum S (2017). The evolution of the host microbiome as an ecosystem on a leash. Nature.

[50] Jousset A, Bienhold C, Chatzinotas A (2017). Where less may be more: how the rare biosphere pulls ecosystems strings. ISME J.

[51] Kaplan A, Zelicha H, Tsaban G (2019). Protein bioavailability of Wolffia globosa duckweed, a novel aquatic plant - a randomized controlled trial. Clin Nutr.

[52] Yaskolka Meir A, Tuohy K, von Bergen M (2021). The metabolomic-gut-clinical axis of Mankai plant-derived dietary polyphenols. Nutrients.

[53] Graham HN (1992). Green tea composition, consumption, and polyphenol chemistry. Prev Med (Baltim).

[54] Appenroth K-J, Sree KS, Bog M, et al. Nutritional value of the duckweed species of the genus Wolffia (Lemnaceae) as human food. Front Chem. 2018;6. 10.3389/fchem.2018.00483 PMC6215809 30420949

[55] De Filippo C, Cavalieri D, Di Paola M (2010). Impact of diet in shaping gut microbiota revealed by a comparative study in children from Europe and rural Africa. Proc Natl Acad Sci U S A.

[56] Vangay P, Johnson AJ, Ward TL (2018). US immigration westernizes the human gut microbiome. Cell.

[57] De Vadder F, Kovatcheva-Datchary P, Zitoun C, Duchampt A, Bäckhed F, Mithieux G (2016). Microbiota-produced succinate improves glucose homeostasis via intestinal gluconeogenesis. Cell Metab.

[58] Cani PD (2018). Human gut microbiome: hopes, threats and promises. Gut.

[59] Michael DR, Jack AA, Masetti G (2020). A randomised controlled study shows supplementation of overweight and obese adults with lactobacilli and bifidobacteria reduces bodyweight and improves well-being. Sci Rep.

[60] Koeth RA, Wang Z, Levison BS (2013). Intestinal microbiota metabolism of l-carnitine, a nutrient in red meat, promotes atherosclerosis. Nat Med.

[61] Tang WHW, Kitai T, Hazen SL. Gut microbiota in cardiovascular health and disease. Physiol Rev. 2017:859–904. 10.1161/CIRCRESAHA.117.309715 PMC5390330 28360349

[62] Rinott E, Yaskolka Meir A, Tsaban G, et al. The effects of the Green-Mediterranean diet on cardiometabolic health are linked to gut microbiome modifications: online resource [https://www.ebi.ac.uk/ena/browser/view/PRJEB49146]. ENA PRJEB49146. 10.1186/s13073-022-01015-z PMC8908597 35264213

[63] Rinott E, Yaskolka Meir A, Tsaban G, et al. The effects of the Green-Mediterranean diet on cardiometabolic health are linked to gut microbiome modifications: online resource [https://www.ebi.ac.uk/ena/browser/view/PRJEB49147]. ENA. PRJEB49147. 10.1186/s13073-022-01015-z PMC8908597 35264213

