# Supplementary material for: The effects of the Green-Mediterranean diet on cardiometabolic health are linked to gut microbiome modifications: a randomized controlled trial
Source: Genome Med. 2022 Mar 10;14:29. doi: 10.1186/s13073-022-01015-z (PMC8908597; doi:10.1186/s13073-022-01015-z)
Supplement: Supplementary file 1 — Additional file 1. Methods – Exclusion criteria, Physical activity protocol, Lifestyle sessions and motivation techniques, Blood sample analysis, 16s rRNA sequencing pipeline, Metagenomics analysis BoosterShot pipeline. [file 13073_2022_1015_MOESM1_ESM.docx]

**Additional file 1: Methods**

Exclusion criteria

Exclusion criteria were an inability to partake in physical activity, serum creatinine level≥2mg/dL, disturbed liver function, a major illness that might require hospitalization, pregnancy or lactation for women, presence of active cancer or undergoing chemotherapy either at present or in the prior three years, participation in another trial, chronic treatment with warfarin (given its interaction with vitamin K), and being implanted with a pacemaker or platinum implant (due to inability to undergo magnetic resonance imaging included in the study design).

Physical activity protocol

The aerobic effort increased gradually, starting with 20 minutes of aerobic training at 65% maximum heart rate, and increased to 45-60 minutes of aerobic training at 80% of maximum heart rate. The full workout program included 45-60 minutes of aerobic training 3-4 times/week; resistance training starting with one set of weights corresponding to 60% of the maximum weight, eventually reached the use of two sets of weights corresponding to 80% of the maximum weight. The resistance training included leg extensions, leg curls, squats, lateral pull-downs, push-ups, shoulder presses, elbow flexions, triceps extensions, and bent leg sit-ups.

Lifestyle sessions and motivation techniques

The lifestyle interventions included 90-minute nutritional and physical activity sessions in the workplace with multidisciplinary guidance (physicians, clinical dietitians, and fitness instructors). These sessions were held every week during the first month, once a month, over the following five months. All the lifestyle educational programs were provided at the same intensity to all three groups. To keep the participants motivated, text messages with relevant information for each assigned intervention group were sent on fixed time intervals. In addition, a website listing all nutritional and physical activity information needed by the participants to continue with the intervention was accessible to the participants according to their intervention group.

Blood sample analysis

Serum total cholesterol (TC;Coefficient-of-variation (CV), 1.3%), HDL-c, low-density-lipoprotein-cholesterol (LDL-c), and TG (CV, 2.1%) were determined enzymatically with a Cobas-6000 automatic analyzer (Roche). Plasma levels of high-sensitivity C-reactive protein (hsCRP) were measured by ELISA (DiaMed;CV, 1.9%). Plasma glucose levels were measured by Roche GLUC3 (hexokinase method). Plasma insulin levels were measured with an enzyme immunometric assay (Immulite automated analyzer, Diagnostic Products; CV, 2.5%). The homeostatic model of insulin resistance (HOMA IR) was calculated as follows: insulin(μIU/ml)×glucose(mg/dl)/405[19]. All biochemical analyses were performed at the University of Leipzig, Germany.

16s rRNA sequencing pipeline

DNA Extraction:

Fecal microbiota DNA was extracted using QIAamp PowerFecal DNA Kit (Qiagen) and a FastPrep-24™bead beater (MP Biomedicals). DNA quantity and quality was assessed spectrophotometrically by NanoDrop™ (ThermoFisher Scientific).

Library Preparation & Sequencing:

For 16S rRNA sequencing amplification of total genomic faecal DNA was carried out using the specific bacterial primer set 341F (5’ CCTACGGGNGGCWGCAG 3’) and 806R (5’ GACTACNVGGGTWTCTAATCC 3’) with overhang Illumina adapters, targeting a ~460-bp fragment of the 16S rRNA variable region V3-V4.^2,3^

PCR amplification of each sample was carried out using 25 µl reactions with 0.2 µM of each primer and 12.5 ng template DNA, and employing KAPA HiFi HotStart ReadyMix. PCR amplification was carried out using a GeneAmp PCR System 9700 (Thermo Fisher Scientific) with the following steps: one cycle at 94°C for 5 minutes, 30 cycles at 95°C for 30 seconds, 55°C for 30 seconds, 72°C for 30 seconds and one final elongation step at 72°C for 5 minutes. The PCR products were checked on 1.5% agarose gel and cleaned from free primers and primer dimer using the Agencourt AMPure XP system (Beckman Coulter, Brea, CA, USA) following the manufacturer’s instructions. Subsequently dual indices and Illumina sequencing adapters Nextera XT Index Primer (Illumina) were attached by 7 cycles PCR (16S Metagenomic Sequencing Library Preparation, Illumina).

The final libraries were quantified using the Quant-IT PicoGreen dsDNA assay kit (Thermo Fisher Scientific) by the Synergy2 microplate reader (Biotek), then libraries were pooled in an equimolar way and analysed on a Typestation 2200 platform (Agilent Technologies, Santa Clara, CA, USA). Barcoded library were sequenced on Illumina® MiSeq (PE300) platform (MiSeq Control Software 2.0.5 and Real-Time Analysis software 1.16.18). Sequences with expected error rate >1.5% were removed from analysis.

Metagenomics analysis BoosterShot pipeline

DNA Extraction:

Samples were extracted using MO Bio PowerFecal ([Qiagen](https://www.qiagen.com/us/shop/sample-technologies/dna/genomic-dna/qiaamp-powerfecal-dna-kit/?cmpid=COECM_MOBIO_Branded_1707_SEA_PowerFecal__&kwid=+power%20+fecal&gclid=EAIaIQobChMIkuCfoo2s3gIVQbXACh1YZATFEAAYASAAEgIgu_D_BwE&clear=true#orderinginformation)) automated for high throughput on QiaCube ([Qiagen](https://www.qiagen.com/us/shop/automated-solutions/sample-preparation/qiacube/#orderinginformation)), with bead beating in 0.1mm glass bead plates.

DNA Quantification:

Samples were quantified with Qiant-iT Picogreen dsDNA Assay ([Invitrogen](https://www.thermofisher.com/order/catalog/product/P7589)).

Library Preparation & Sequencing

Libraries were prepared with a procedure adapted from the Nextera Library Prep kit ([Illumina](https://www.illumina.com/products/by-type/sequencing-kits/library-prep-kits/nextera-dna.html)). Libraries were sequenced on an Illumina NextSeq using single-end 1 x 145 reads with a NextSeq 500/550 High Output v2 kit ([Illumina](https://www.illumina.com/products/by-type/sequencing-kits/cluster-gen-sequencing-reagents/nextseq-series-kits-v2.html)).

Sequence Quality Control:

DNA sequences were filtered to remove low quality (Q-Score < 20) reads, and for length (< 50), and adapter sequences were trimmed using [cutadapt](http://cutadapt.readthedocs.io/en/stable/guide.html). Fastq files were converted a single fasta using [shi7](https://github.com/knights-lab/shi7).

OTU Picking:

DNA sequences were aligned to a curated database containing all representative genomes in [RefSeq](https://www.ncbi.nlm.nih.gov/refseq) for bacteria with additional manually curated strains. Alignments were made at 98% identity against all reference genomes. Each input sequence was compared to each reference sequence in CoreBiome’s Venti database using full gapped alignment with [Burst](https://github.com/knights-lab/BURST). Ties were broken by minimizing the overall number of unique gene hits. For taxonomy assignment, each input sequence was assigned the lowest common ancestor that was consistent across at least 80% of all reference sequences tied for best hit. The number of counts for each taxon was then normalized to the average genome length. Species accounting for less 1X10^-6^ of all species-level markers were discarded. Samples with fewer than 1,000 sequences were also discarded. The normalized and filtered tables were used for all downstream analyses.

Functional Genome Content:

Functional groups were observed directly using Kyoto Encyclopedia of Genes and Genome Orthology groups ([KEGG KOs](https://www.genome.jp/kegg/)) by alignment against a gene database derived from the strain database used above.
