# Supplementary material for: The effects of the Green-Mediterranean diet on cardiometabolic health are linked to gut microbiome modifications: a randomized controlled trial
Source: Genome Med. 2022 Mar 10;14:29. doi: 10.1186/s13073-022-01015-z (PMC8908597; doi:10.1186/s13073-022-01015-z)
Supplement: Supplementary file 2 — Additional file 2: Table S1. Outline of the lifestyle interventions. [file 13073_2022_1015_MOESM2_ESM.docx]

**Additional file 2: Table S1 - Outline of the lifestyle interventions**

| Green-MED | | MED | HDG |  |
| --- | --- | --- | --- | --- |
| 18 months free gym membership  45-60 minutes of aerobic training + resistance training, 3-4 times/week. | | | | Physical activity |
| 18-months group sessions in the workplace, weekly for the first month, and monthly thereafter. | | | | Lifestyle group sessions |
| Limit dietary cholesterol, trans-fat, saturated-fat, sugars, and salt and increase intake of vegetables | | | | General dietary guidance |
| 1500-1800 kcal/day for men, 1200-1400 kcal/day for women | | | Guidelines for a healthy MED diet with no specific recipes or calorie restriction | Energy, kcal/day |
| ~40% mainly PUFA and MUFA | | |  | Total fat, % of daily consumption |
| Less than 40 gr/day in the first 2 months with increased gradual intake for up to 80 gr/day | | |  | Carbohydrates, gr/day |
| Less/Avoid red and processed meats. Reduced poultry intake | | |  | Specific recommendations |
| +1240 mg/day  [source: provided walnuts (28 g/day), green tea (3-4 cups/day), Wolffia globosa duckweed (Mankai) shake (100 g/day frozen cubes)] | +440 mg/day  [source: provided  walnuts (28g/day] | |  | Polyphenols, mg/day |
