# Supplementary material for: The effects of the Green-Mediterranean diet on cardiometabolic health are linked to gut microbiome modifications: a randomized controlled trial
Source: Genome Med. 2022 Mar 10;14:29. doi: 10.1186/s13073-022-01015-z (PMC8908597; doi:10.1186/s13073-022-01015-z)
Supplement: Supplementary file 3 — Additional file 3: Table S2. Characteristics of the study population. [file 13073_2022_1015_MOESM3_ESM.docx]

**Additional file 3: Table S2 - Characteristics of the study population**

|  | Healthy dietary guidelines | Mediterranean diet | Green-Mediterranean diet |
| --- | --- | --- | --- |
| ﻿Subjects – no. | 96 | 95 | 95 |
| Fecal sample follow-up  at 6m - % | 86.5 | 92.7 | 82.1 |
| Age ﻿- yr | 51.34 (10.50) | 51.74 (10.41) | 50.68 (10.87) |
| Male sex - % | 85 ( 88.5) | 84 ( 87.5) | 85 ( 89.5) |
| Weight - kg | 92.53 (14.50) | 94.53 (13.63) | 93.68 (14.97) |
| ﻿Waist circumference- cm |  |  |  |
| Male | 43.52 (9.91) | 46.18 (10.04) | 43.11 (10.80) |
| Female | 59.39 (13.21) | 54.41 (15.71) | 61.62 (14.02) |
| FPG – mg/dl | 102.07 (17.74) | 100.69 (13.42) | 103.07 (19.74) |
| Fasting insulin- microU/ml | 15.28 (9.07) | 14.46 (7.03) | 14.21 (7.40) |
| HOMA-IR | 3.97 (2.76) | 3.65 (1.92) | 3.66 (2.20) |
| LDL cholesterol- mg/dL | 126.59 (32.56) | 126.68 (31.27) | 123.33 (28.90) |
| HDL cholesterol- mg/dL |  |  |  |
| Male | 43.52 (9.91) | 46.18 (10.04) | 43.11 (10.80) |
| Female | 59.39 (13.21) | 54.41 (15.71) | 61.62 (14.02) |
| Total Cholesterol | 192.10 (36.11) | 193.67 (31.70) | 185.61 (31.38) |
| Leptin – ng/ml | 13.12 (10.63) | 14.98 (12.45) | 13.79 (11.16) |
| Ghrelin – ng/ml | 515.48 (231.11) | 488.71 (184.21) | 500.14 (198.08) |
| Systolic BP - mmHg | 130.10 (14.38) | 130.24 (12.48) | 130.57 (15.19) |
| ﻿Diastolic BP - mmHg | 80.01 (11.32) | 81.78 (8.86) | 81.49 (10.38) |
| Mean arterial pressure -mmHg | 96.71 (11.62) | 97.93 (9.02) | 97.85 (10.86) |
| Oral glycemic control (%) | 5 (5.2) | 4 (4.2) | 7 (7.4) |
| Antihypertensive(%) | 13 ( 13.5) | 11 ( 11.5) | 16 ( 16.8) |
| Lipid lowering (%) | 11 ( 11.5) | 8 (8.3) | 14 ( 14.7) |
| Anti Platelet (%) | 6 (6.2) | 3 (3.1) | 9 (9.5) |
| Smoking(%) | 18 ( 18.9) | 11 ( 11.7) | 16 ( 16.8) |

Baseline characteristics of DIRECT PLUS participants with available baseline fecal sample. Values are presented as means (standard deviation) for continues variables and total number (percent) for categorical variables. No significant differences were observed between intervention groups in the measured baseline characteristics. ﻿BMI, body mass index; BP, blood pressure; FPG, fasting plasma glucose; HDL- C, high- density lipoprotein cholesterol; LDL- C, low- density lipoprotein cholesterol; HOMA- IR, homoeostatic model assessment of insulin resistance.
