# Supplementary material for: The effects of the Green-Mediterranean diet on cardiometabolic health are linked to gut microbiome modifications: a randomized controlled trial
Source: Genome Med. 2022 Mar 10;14:29. doi: 10.1186/s13073-022-01015-z (PMC8908597; doi:10.1186/s13073-022-01015-z)

Flow Diagram of the DIRECT-PLUS trial

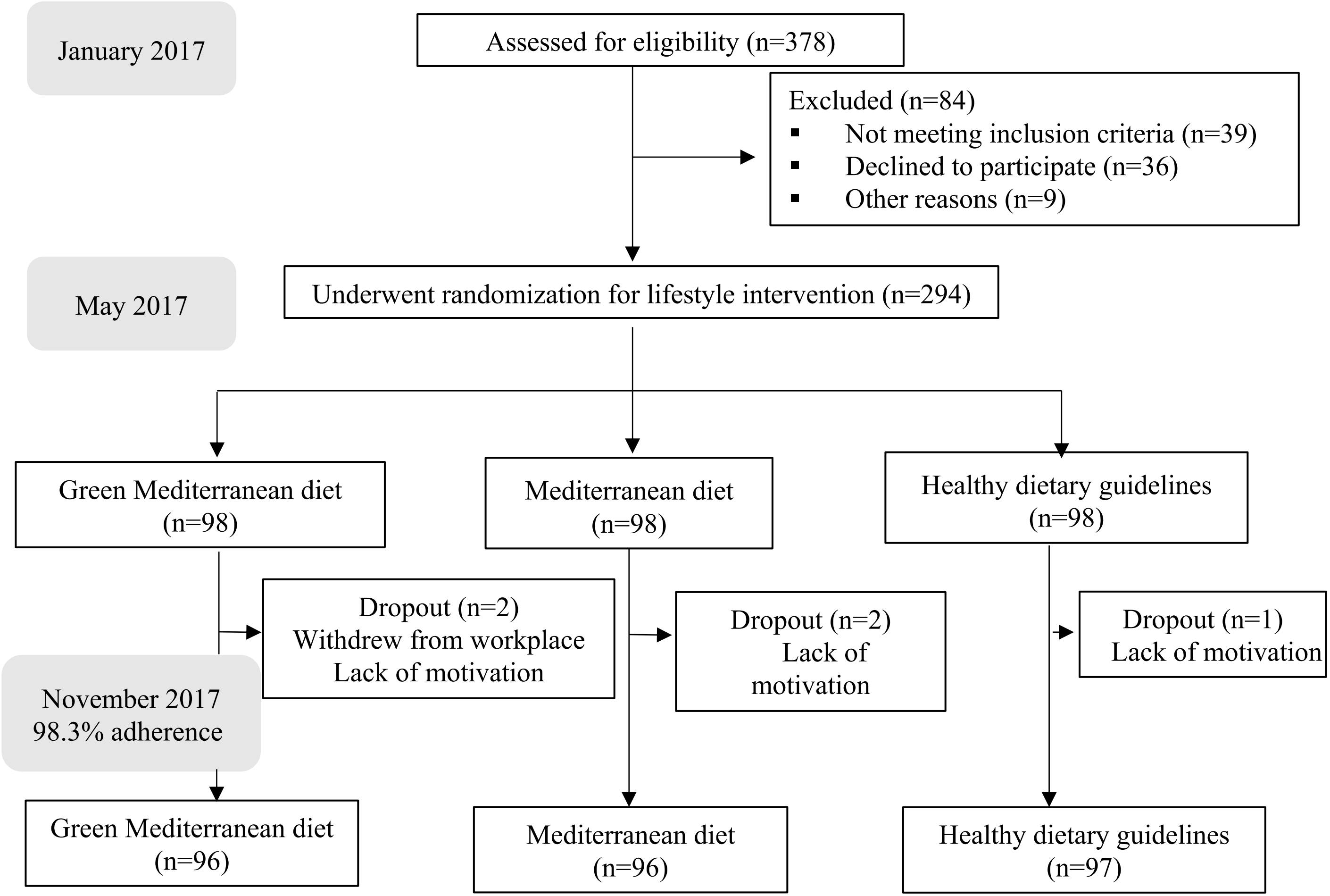

Supplement: Supplementary file 4 — Additional file 4: Figure S1. Flow Diagram of the DIRECT-PLUS trial. [file 13073_2022_1015_MOESM4_ESM.pdf]
