# Supplementary material for: The effects of the Green-Mediterranean diet on cardiometabolic health are linked to gut microbiome modifications: a randomized controlled trial
Source: Genome Med. 2022 Mar 10;14:29. doi: 10.1186/s13073-022-01015-z (PMC8908597; doi:10.1186/s13073-022-01015-z)
Supplement: Supplementary file 5 — Additional file 5. Contains Figures S2 and S3. [file 13073_2022_1015_MOESM5_ESM.docx]

**Additional file 4: Figure S2**


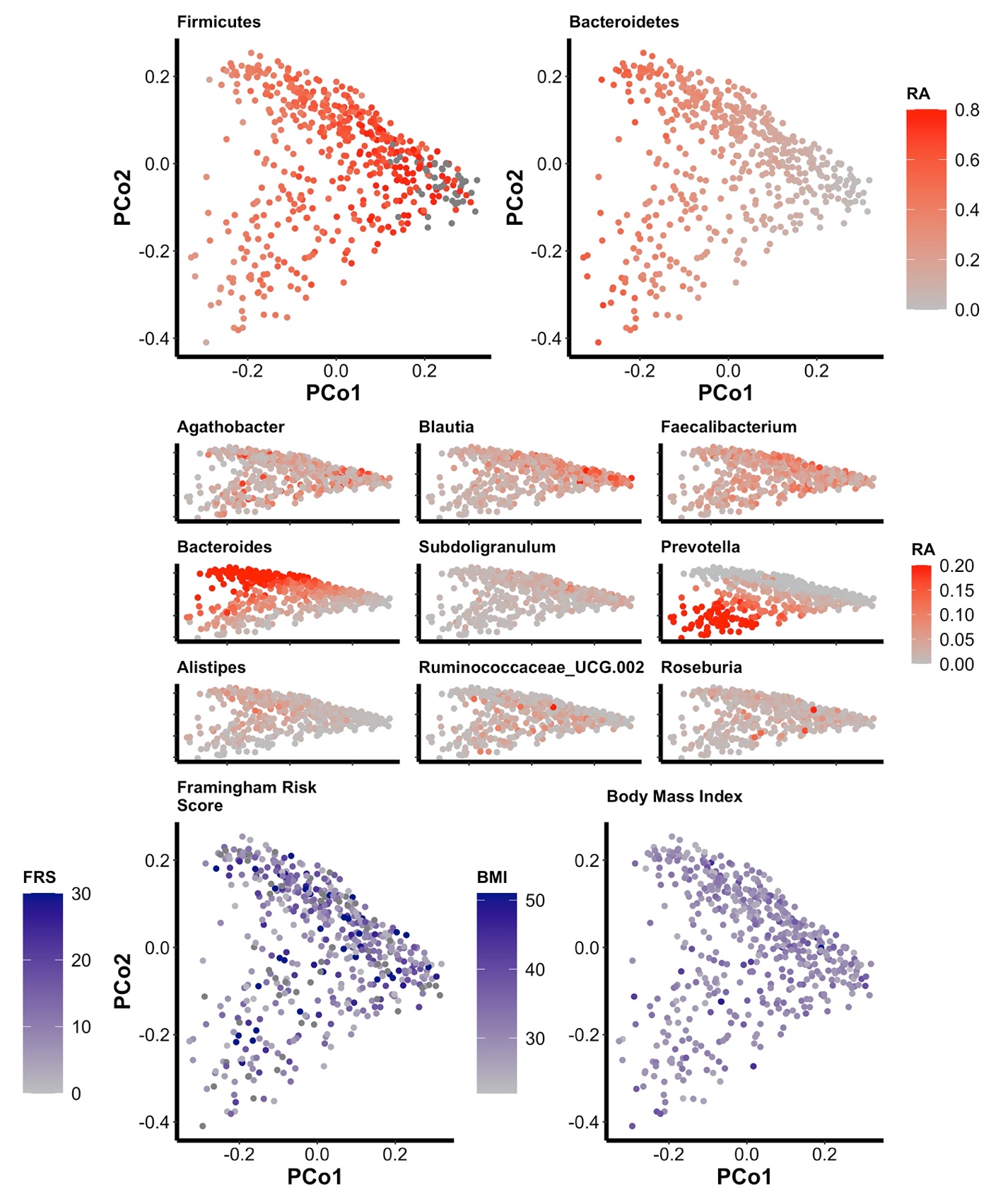
**Figure S2.** Principal coordinate analysis of Weighted UniFrac dissimilarity colored in correspondence to the relative abundance of the 2 most abundant Phylum, 9 most abundant Genus, Framingham risk score and Body Mass Index.

**Additional file 4: Figure S3**


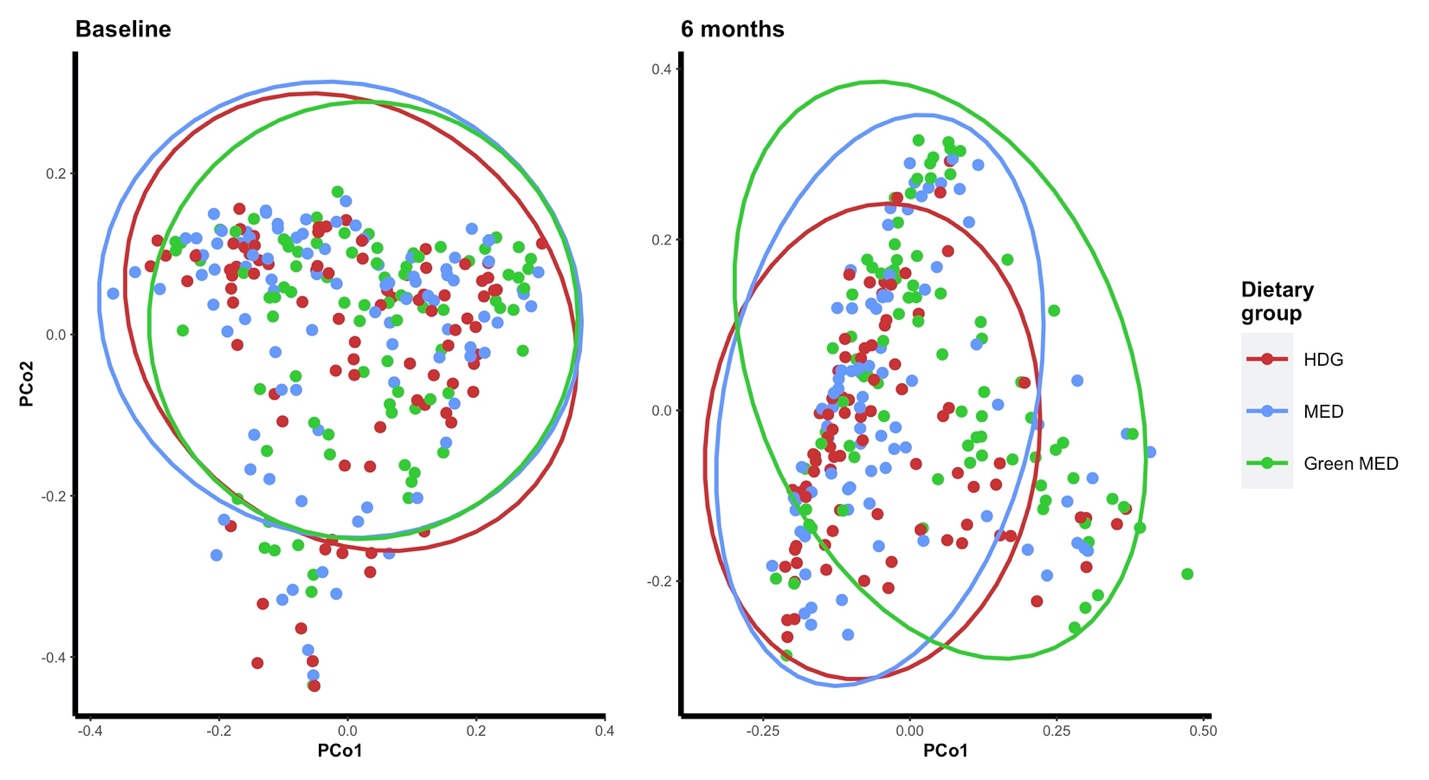


**Figure S3.** Principal coordinate analysis of Weighted UniFrac dissimilarity at baseline (left) and 6 months colored by lifestyle intervention group
